# Dynamic changes of metabolic characteristics in neonatal intrahepatic cholestasis caused by citrin deficiency

**DOI:** 10.3389/fmolb.2022.939837

**Published:** 2022-08-24

**Authors:** Ting Zhang, Shasha Zhu, Haixia Miao, Jianbin Yang, Yezhen Shi, Yuwei Yue, Yu Zhang, Rulai Yang, Benqing Wu, Xinwen Huang

**Affiliations:** ^1^ Department of Genetics and Metabolism, Children’s Hospital of Zhejiang University School of Medicine, National Clinical Research Center for Child Health, Hangzhou, China; ^2^ Department of Technical Support, Zhejiang Biosan Biochemical Technologies Co. Ltd., Hangzhou, China; ^3^ Department of Neonatology, Children’s Medical Center, University of Chinese Academy of Science-Shenzhen Hospital, Shenzhen, China

**Keywords:** NICCD, newborn-screen group, clinical diagnosed group, metabolic characteristics, progression, prognosis

## Abstract

**Introduction:** Neonatal intrahepatic cholestasis caused by citrin deficiency (NICCD) is a pan-ethnic complicated inborn error of metabolism but the specific mechanism is not fully understood.

**Methods:** A total of 169 patients with NICCD who have biallelic pathogenic *SLC25A13* variants detected by targeted next-generation sequencing were collected. They were divided into the “Newborn-screen Group” and “Clinical diagnosed Group” depending on the newborn screening results. Amino acid and acylcarnitine profiles were measured by MS/MS. The total bile acids, blood amino acids and acylcarnitines, general biochemistry, blood count, and coagulation parameters were monitored every 2–3 months. We compared the differences in metabolic indices and their dynamic changes between these two groups. The Mann–Whitney test and orthogonal partial least squares discrimination analysis (OPLS-DA) were used for statistical analysis.

**Results:** At the onset of NICCD, we found that the “Clinical diagnosed Group” had higher levels of intermediate products of the urea cycle, free carnitine, and short-chain and long-chain acylcarnitines than those in the “Newborn-screen Group,” but the levels of ketogenic/glucogenic amino acids and several medium-chain acylcarnitines were lower. Furthermore, concentrations of direct bilirubin, total bile acid, lactate, prothrombin time, and several liver enzymes were significantly higher while total protein, amylase, and hemoglobin were lower in the “Clinical diagnosed Group” than in the “Newborn-screen Group.” Dynamic change analysis showed that direct bilirubin, albumin, arginine, and citrulline were the earliest metabolic derangements to reach peak levels in NICCD groups, followed by acylcarnitine profiles, and finally with the elevation of liver enzymes. All abnormal characteristic metabolic indicators in the “Newborn-screen Group” came back to normal levels at earlier ages than the “Clinical diagnosed Group.” c.852_855del (41.2%), IVS16ins3kb (17.6%), c.615 + 5G>A (9.6%), 1638_1660dup (4.4%), and c.1177 + 1G>A (3.7%) accounted for 76.5% of all the mutated *SLC25A13* alleles in our population.

**Conclusion:** Argininosuccinate synthesis, gluconeogenesis, ketogenesis, fatty acid oxidation, liver function, and cholestasis were more severely affected in the “Clinical diagnosed Group.” The “Newborn-screen Group” had a better prognosis which highlighted the importance of newborn screening of NICCD.

## Introduction

Citrin is an aspartate/glutamate carrier (AGC) isoform-2 of the mitochondrial inner membrane expressed in the liver, kidney, and heart (Palmieri et al., 2001). Citrin deficiency is caused by biallelic pathogenic variants in the *SLC25A13* gene (Saheki and Kobayashi, 2002). There are three age-dependent phenotypes described: neonatal intrahepatic cholestasis caused by citrin deficiency (NICCD, OMIM#605814), failure to thrive and dyslipidemia caused by citrin deficiency (FTTDCD), and the adult-onset citrullinemia type II (CTLN2, OMIM#603471). NICCD causes intrahepatic cholestasis and manifests clinically with citrullinemia, hyperammonemia, hypoproteinemia, galactosemia, and hypoglycemia (Saheki and Kobayashi, 2002; Ohura et al., 2007). FTTDCD occurs at the post-NICCD but pre-CTLN2 stage. After the NICCD period, some individuals may progress to FTTDCD, and only a few patients may develop severe CTLN2 symptoms decades later (Kobayashi et al., 2003).

NICCD usually resolves spontaneously within the first year after appropriate treatment. However, very few subjects develop severe hepatic dysfunction which may require liver transplantation or die before the transplantation (Shigeta et al., 2010; Song et al., 2011; Zhang et al., 2015). The mortality of NICCD was found to be associated with lower platelet count, lower levels of gamma-glutamyl transpeptidase, total cholesterol, blood citrulline, and higher levels of blood ammonia and tyrosine (Abuduxikuer et al., 2019).

The specific mechanism leading to the complicated heterogeneity of NICCD is not fully understood. In the malate-aspartate NADH shuttle (MA shuttle), AGC is important for the transport of reducing the equivalent of NADH into mitochondria and the transport of aspartate to the cytosol. This function can maintain a low and high NADH/NAD^+^ ratio in the cytosol and mitochondria, respectively, which is necessary for the synthesis of urea, proteins, and nucleotides (Saheki et al., 2020). The deficiency of citrin is thought to cause various metabolic abnormalities such as inhibition of glycolysis, gluconeogenesis, and urea synthesis based on current research studies (Okano et al., 2019).

At present, tandem mass spectrometry (MS/MS) is widely used in newborn screening since it can simultaneously test 11 amino acids, 30 acylcarnitines, free carnitine, and succinylacetone (Wang et al., 2019). The elevated citrulline level is the primary screening marker for NICCD, but the citrulline level may not be elevated at the age of 3–7 days which limited the performance of MS/MS analysis and lead to several missed cases (Lin et al., 2020). Several methods such as increasing a secondary indicator or combining high throughput iPLEX genotyping assay can identify an additional subgroup of patients with NICCD that are undetectable by conventional newborn screening (Tang Chenfang et al., 2019; Lin et al., 2020). The NICCD patients diagnosed by newborn screening will be timely treated with a dietary management of lactose-free and/or medium-chain triglyceride-enriched (LF/MCT) formula (Ohura et al., 2007). However, some of the missed NICCD cases may develop more severe and acute symptoms including cholestasis and hepatic dysfunction during infancy.

In this study, we studied the differences in metabolic indices and their dynamic changes between the “Newborn-screen Group” and the “Clinical diagnosed Group” to get a better understanding of disease progression and search for potential monitoring biomarkers.

## Methods

### Study subjects

A total of 169 patients with NICCD were collected from Children’s Hospital, Zhejiang University School of Medicine, from January 2010 to May 2021. Among them, 51 patients were diagnosed by the newborn screening of 4.2 million Chinese neonates. They were named the “Newborn-screen Group.” The total frequency of NICCD in our cohorts is 1:82352. A total of 118 patients were admitted to the hospital with suspected neonatal hepatitis or biliary atresia and finally diagnosed with NICCD. They were named the “Clinical diagnosed Group.” Among them, 42 had negative MS/MS newborn screening results and 76 patients had not undergone newborn screening. All cases were confirmed by mutation analysis of the *SLC25A13* gene.

This study was approved by the Ethical Committee of Children’s Hospital, Zhejiang University School of Medicine (reference number: 2020-IRBAL-035). Written informed consent was obtained from the parents of all infants for the collection of samples and publication of medical data.

### Metabolic index detection and molecular testing

The age of the first detection for MS/MS of the “Newborn-screen Group” and “Clinical diagnosed Group” was 0.12 ± 0.03 months and 2.68 ± 1.45 months, respectively. In newborn screening, dried blood spot (DBS) samples were collected by the heel-stick method and spotted on Whatman 903 filter paper for the neonates at 3–7 days after birth. In clinical screening, DBS samples were collected by the heel-stick method or digit for the patients with intrahepatic cholestatic. Amino acid and acylcarnitine profiles were measured by MS/MS with the NeoBase Non-derivatized MSMS Kit (PerkinElmer, Finland). In brief, a 100 μl working solution containing internal standards was added in U bottom plates. After vibrating at 700 rpm and incubating for 45 min at 45°C, 75 μl liquor was transferred into V bottom plates. After 2 h standing at room temperature, 25 μl liquor was injected into tandem mass spectrometry for metabolic analyses. Two levels of internal quality controls including low and high were used for quality control (Wang et al., 2019).

The age of the first detection for general biochemistry, blood count, and coagulation parameters of the “Newborn-screen Group” and “Clinical diagnosed Group” was 1.53 ± 1.03 months and 2.78 ± 1.85 months, respectively. Subsequently, the biochemistry data, blood amino acids, and acylcarnitines were monitored every 2–3 months for dynamic change analysis. After the clinical manifestations and related indicators returned to normal, a follow-up was performed every 6 or 12 months.

Genomic DNA was extracted from probands and their parents. Targeted next-generation sequencing was performed with a genetic diagnosis panel of hereditary metabolic diseases covering 306 genes (Wang et al., 2019). The genealogies of suspected mutations were determined *via* Sanger sequencing. Long-range PCR analysis was performed to screen for the 3 kb insertion mutation in intron 16 (IVS16ins3kb) (Chong et al., 2018).

### Treatment

Treatment of NICCD patients was performed with several aspects as follows. Breast milk and ordinary formula were replaced by lactose-free milk and medium-chain triglyceride (MCT)-added milk. Ursodeoxycholic acid (sodium salt) was used to improve the cholestatic features and arginine was used to reduce ammonia. Dietary treatment based on low-carbohydrate and lipid and protein-rich were introduced after adding complementary foods. Fat-soluble vitamins were supplemented.

### Statistical analysis

The reference intervals (RIs) of metabolic indices were determined corresponding to age-matched controls. The positive rate was calculated for each indicator when the value is higher or lower than the reference intervals.

SPSS 22.0 software was used for statistical analysis of metabolic indices at the age of the first detection between the “Newborn-screen Group” and “Clinical diagnosed Group.” Continuous variables were described as mean ± standard deviation (SD). The Shapiro–Wilk normality test was performed to determine whether each continuous variable is normally distributed or not. Continuous variables with normal distribution were compared by Student’s t-test, while Mann–Whitney tests were used to compare variables that were not normally distributed. *p*-values of less than 0.05 were considered statistically significant (He et al., 2021). The orthogonal partial least-squares-discriminant analysis (OPLS-DA) was used to identify different indicators between the “Newborn-screen Group” and “Clinical diagnosed Group.” The variable importance in projection (VIP) generated in OPLS-DA represents the contribution to the discrimination of each metabolite between groups. Variables with a VIP >1 were significantly different (Li et al., 2018).

The dynamic change analysis of typical indicators between the “Newborn-screen Group” and “Clinical diagnosed Group” was shown further by drawing boxplots with R 4.0.5 software. GraphPad Prism 8 software was used for drawing the figure for sequential order analysis.

## Results

### Metabolic characteristic analysis in the “newborn-screen group” and “clinical diagnosed group” at the onset of neonatal intrahepatic cholestasis caused by citrin deficiency

The positive rates were used for reflecting the metabolic characteristics of the two groups. They were calculated by the reference intervals corresponding to age-matched controls. In the amino acid profiles, the “Newborn-screen Group” was featured with elevated concentrations of Cit, Met, Phe, and Tyr (positive rate >20%), while the “Clinical diagnosed Group” was characterized by elevated levels of Cit, Arg, Met, Tyr, and Orn (positive rate >40%) (Supplementary Table S1). In acylcarnitine profiles, the majority of neonates in the “Newborn-screen Group” did not present with abnormal results. However, a fairly large number of infants (>30%) presented with elevated concentrations of C14, C16, C16:1, C18:1, and C18:2 (Supplementary Table S1).

The biochemical data in the two groups showed similar evidence of intrahepatic cholestasis and liver damage. In more than 50% of patients, the levels of bilirubin (TBil, DBil, and IBil), total bile acid (TBA), gamma-glutamyl transpeptidase (GGT), alkaline phosphatase (ALP), alpha-fetoprotein (AFP), lactate, and ammonia were significantly elevated than the normal range, while the levels of total protein (TP), albumin globulin (ALB), globulin (GLB), cholinesterase (CHE), and amylase were significantly lower than the normal range in the two groups (Table 1).

**TABLE 1:** Biochemical indices of the “Newborn-screen Group” and “Clinical diagnosed Group” at first detection.

Indices	Newborn-screen group (average ± SD, positive rate)	Reference intervals for 10–30 days	Clinical diagnosed group (average ± SD, positive rate)	Reference intervals for 1–12 months
TP (g/L)↓^a^	44.02 ± 6.87 (62.2%)	41.0–63.0	46.13 ± 8.26 (91%)	57.0–80.0
ALB (g/L)↓	30.29 ± 5.55 (54.1%)	28.0–44.0	31.8 ± 5.65 (58.56%)	32.0–52.0
GLB (g/L)↓	13.72 ± 2.55 (100.00%)	20.0–40.0	14.34 ± 3.94 (90.99%)	20.0–40.0
TBil (umol/L)↑^b^	122.33 ± 69.30 (91.9%)	5.0–21.0	139.88 ± 66.05 (94.59%)	5.0–21.0
DBil** (umol/L)↑	41.79 ± 29.83 (91. 9%)	0–5.1	66.08 ± 38.28 (95.5%)	0–5.1
IBil (umol/L)↑	80.54 ± 51.19 (86.49%)	1.0–20.0	73.87 ± 44.91 (90.09%)	1.0–20.0
ALT** (U/L)↑	27.05 ± 13.84 (10.8%)	5–50	44.83 ± 25.73 (34.23%)	5–50
AST** (U/L)↑	69.73 ± 40.95 (35.1%)	25–75	121.93 ± 63.71 (90.99%)	15–60
CHE (U/L)↓	5124 ± 1589 (70.3%)	5300–11300	4689.98 ± 1373.16 (78.38%)	5300–11300
GGT (U/L)↑	189.81 ± 90.19 (94.59%)	8–57	188.72 ± 99.97 (94.59%)	8–57
ALP (U/L)↑	845.48 ± 438.66 (86.49%)	42–362	952.53 ± 512.09 (90.09%)	42–362
ADA** (U/L)↑	13.27 ± 5.93 (37.84%)	0–15	22.22 ± 12.07 (66.67%)	0–15
TBA** (umol/L)↑	190.58 ± 87.89 (83.78%)	0–12	253 ± 101.73 (95.88%)	0–12
urea** (mmol/L)↓	3.73 ± 1.37 (4%)	1.79–6.43	2.82 ± 1.08 (12.12%)	1.79–6.43
Uric Acid* (mmol/L)↓	207.6 ± 73.6 (16%)	155–357	164.65 ± 63.59 (23.47%)	155–357
LDH** (U/L)↑	250.25 ± 147.75 (4.00%)	180–430	473.94 ± 250.57 (65.98%)	180–430
TG (mmol/L)↑	1.69 ± 1.23 (44.00%)	<1.7	1.65 ± 0.74 (41.24%)	<1.7
TCH* (mmol/L)↑	3.88 ± 1.30 (4.00%)	3–5.7	4.52 ± 1.33 (24.74%)	3–5.7
amylase** (U/L)↓	19.14 ± 17.92 (56%)	28–100	13.2 ± 12.0 (52.58%)	28–100
Lactate** (mmol/L)↑	2.22 ± 1.20 (68.18%)	0.5–1.6	4.2 ± 1.95 (93.68%)	0.5–1.6
AFP (ng/mL)↑	113734 ± 127414 (84.38%)	0–20	201751 ± 465135 (92%)	25–100 (1–3M); 25–100 (>3M)
HB** (g/L)↓	115 ± 17 (31.82%)	170–200	96.93 ± 12.97 (80.85%)	110–155
Ammonia** (umol/L)↑	48.26 ± 34.33 (73.68%)	9–30	85.94 ± 35.03 (95.92%)	9–30
PT (s)↑	14.67 ± 2.64 (38.46%)	9–14	15.94 ± 4.02 (62.5%)	9–14
TT^*^(s)↑	21.65 ± 2.05 (30.00%)	15–22	24.95 ± 3.96 (66.67%)	15–22
APTT (s)↑	48.33 ± 18.67 (92.31%)	23–38	52.55 ± 15.05 (36.78%)	23–38

**means the difference in the two groups is significant at the 0.01 level.

*means the difference in the two groups is significant at the 0.05 level.

^↓a^means the positive rate is calculated for the values lower than the normal range.

^↑b^means the positive rate is calculated for the values higher than the normal range.

TP, total protein; ALB, albumin; GLB, globulin; TBil, total bilirubin; DBil, direct bilirubin; IBil, indirect bilirubin; ALT, alanine aminotransferase; AST, aspartate amino transferase; CHE, cholinesterase; GGT, gamma-glutamyl transpeptidase; ALP, alkaline phosphatase; ADA, adenosine deaminase; TBA, total bile acid; LDH, lactate dehydrogenase; TG, triglyceride; TCH, total cholesterol; AFP, alpha fetoprotein; HB, hemoglobin; PT, prothrombin time; TT, thrombin time; APTT, activate partial thrombin activity time.

**FIGURE 1:**
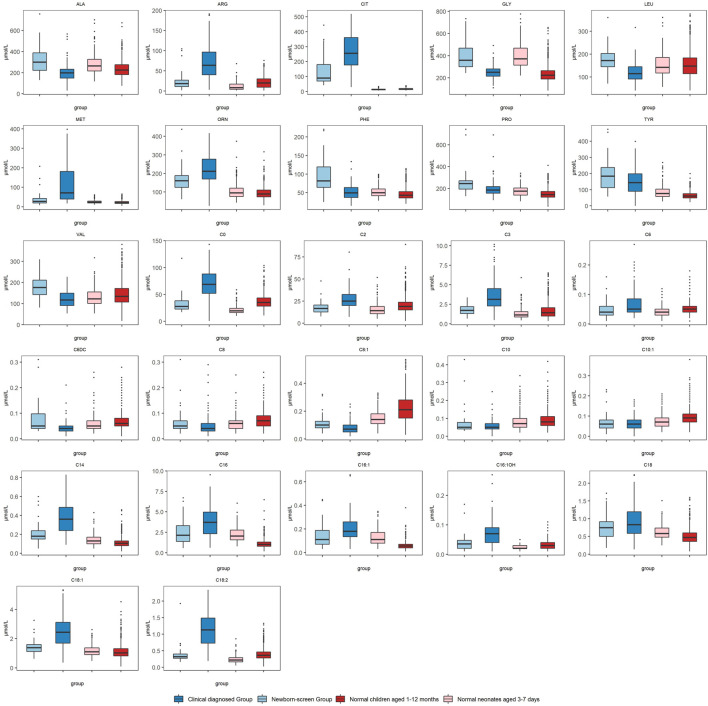
Comparison of MS/MS results in the “Newborn-screen Group” and “Clinical diagnosed Group” at first detection. The “Newborn-screen Group,” the “Clinical diagnosed Group,” “Normal neonates aged 3–7 days,” and “Normal children aged 1–12 months” were distinguished by light blue, dark blue, light red, and dark red boxes, respectively. The horizontal lines on the top, bottom, and inside of the box represent the 75% quantile (Q3), 25% quantile (Q1), and median, respectively. The points distributed outside the upper and lower edges represent the outliers of each indicator.

We compared the metabolic indices in the two groups at first detection which reflected the metabolic status at the onset of NICCD. The “Clinical diagnosed Group” had significantly higher levels of Cit, Arg, Met, and Orn than the “Newborn-screen Group” (Figure 1). However, the concentrations of Phe, Ala, Leu, Pro, and Val were significantly lower than in the “Newborn-screen Group.” In the “Clinical diagnosed Group,” the levels of C0, C2, C3, C14, C16, C16:1, C16:1OH, C18, C18:1, and C18:2 were significantly higher than in the “Newborn-screen Group.” However, the concentrations of C6DC, C8, C8:1, C10, and C10:1 were lower than in the “Newborn-screen Group” (Figure 1).

The concentrations of DBil, alanine aminotransferase (ALT), aspartate amino transferase (AST), adenosine deaminase (ADA), TBA, ammonia, lactate, lactate dehydrogenase (LDH), total cholesterol (TCH), and thrombin time (TT) in the “Clinical diagnosed Group” were significantly higher than those in the “Newborn-screen Group.” However, the concentrations of TP, urea, amylase, and hemoglobin (HB) were significantly lower than in the “Newborn-screen Group” (Table 1).

**FIGURE 2:**
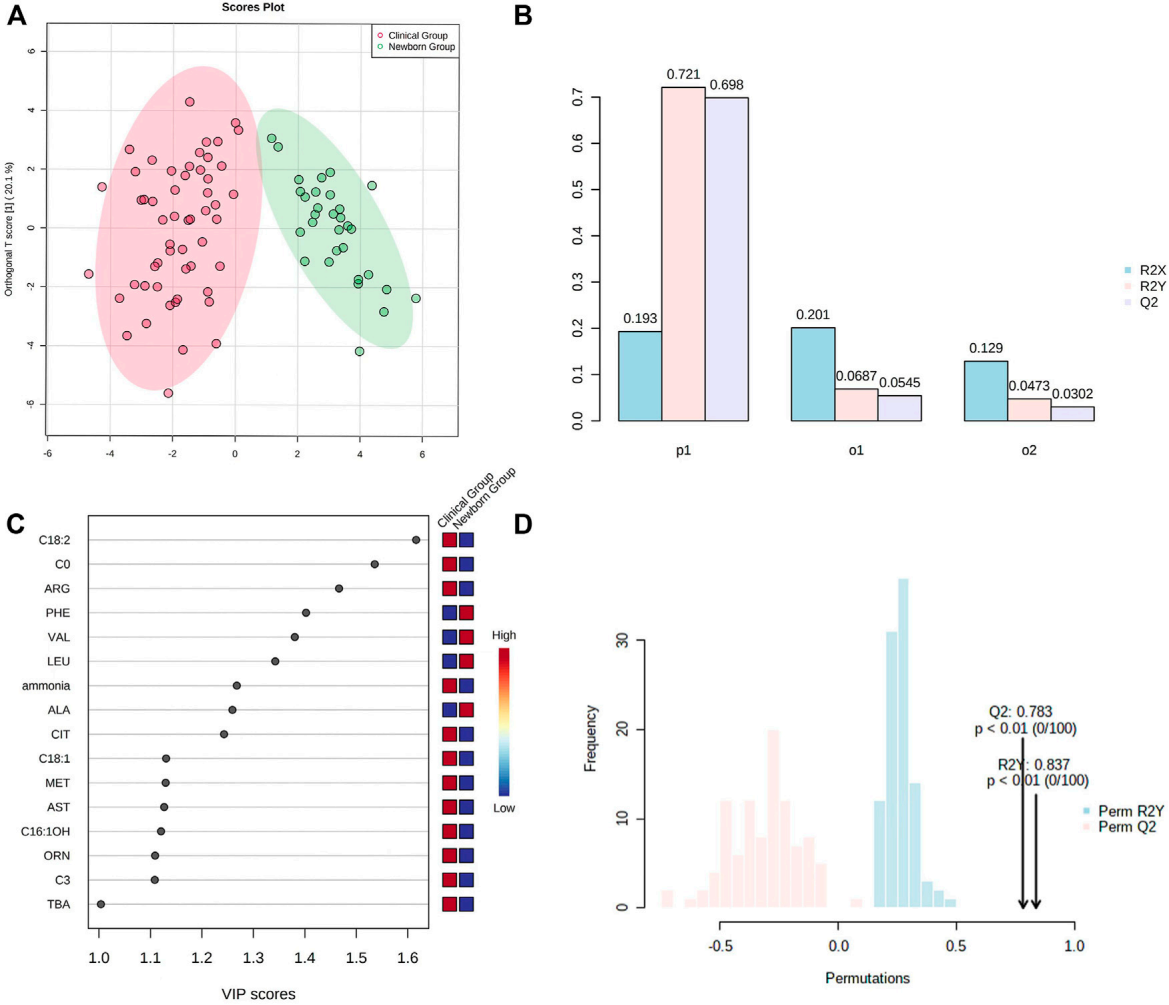
OPSL-DA results of metabolic parameters in the “Newborn-screen Group” and “Clinical diagnosed Group.” **(A)** Score plot of OPSL-DA. The x-axis represents the forecasting principal component and the y-axial represents the orthogonal principal component. Each point in the figure represents a sample. Red is for the “Clinical diagnosed Group” and green is for the “Newborn-screen Group.” **(B)** Inertia column diagram shows R2X, R2Y, and Q2 to evaluate whether the orthogonal components are sufficient. Q2 > 0.5 is considered to be reliable. **(C)** VIP scores of the different indicators are screened. Generally, the VIP value greater than 1 is screened as an important indicator (the 16 indicators are sorted in descending order). **(D)** Model validation diagram. The x-axis represents values of R2X, R2Y, and Q2, while the y-axial represents frequency of the model classification effect. The *p*-value is less than 0.01 which indicates the model is relatively good.

OPLS-DA showed Cit, Arg, Met, Orn, Phe, Ala, Leu, Val, C0, C3, C16:1OH, C18:1, C18:2, ammonia, AST, and TBA representing the most contribution to the discrimination between the “Newborn-screen Group” and “Clinical diagnosed Group” (Figure 2C).

### Dynamic change analysis of typical indicators in the “newborn-screen group” and “clinical diagnosed group” during ages 1–12 months

**FIGURE 3:**
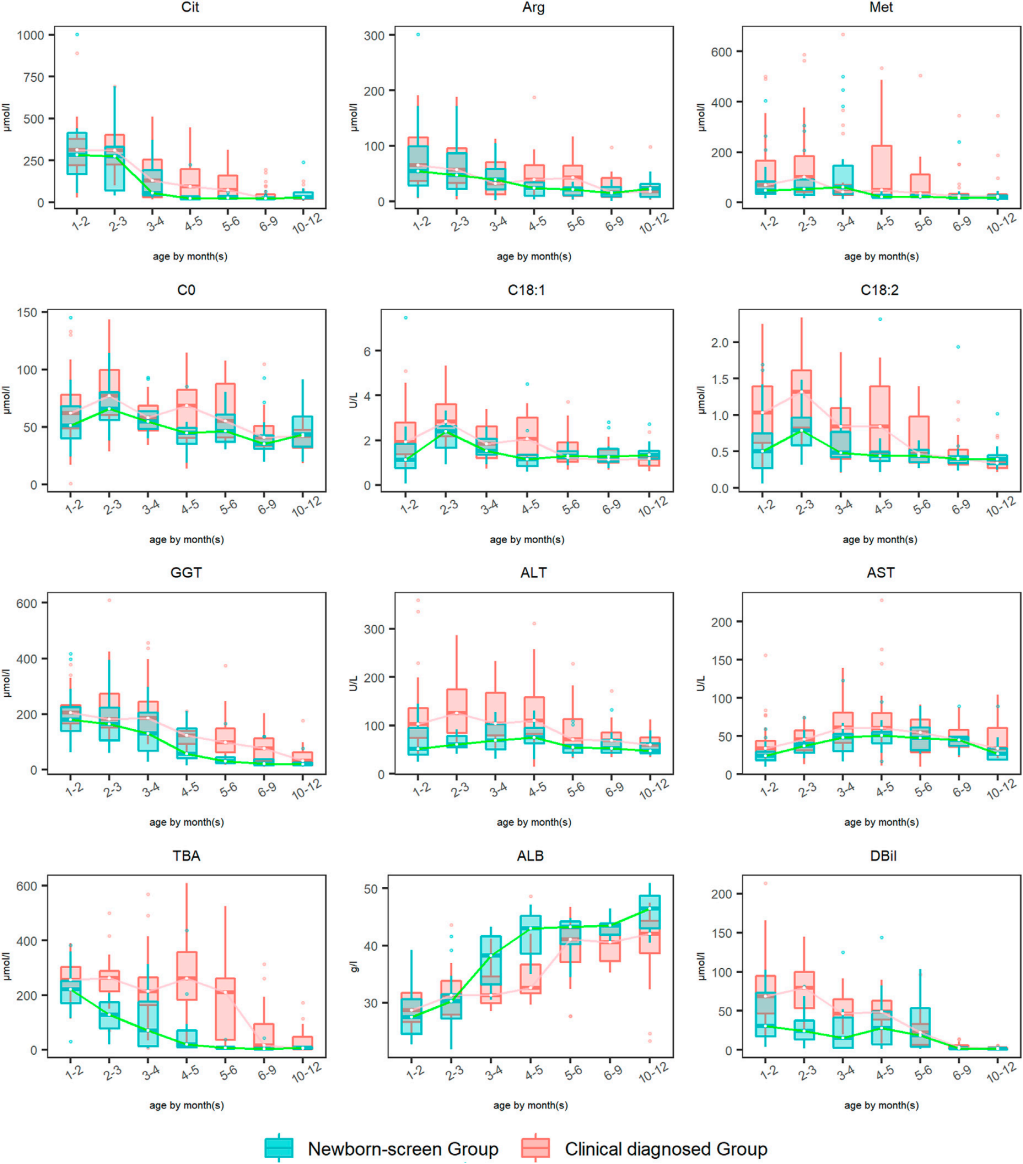
Dynamic change analysis of typical indicators in the “Newborn-screen Group” and “Clinical diagnosed Group” during ages 1–12 months. The “Clinical diagnosed Group” and the “Newborn-screen Group” were distinguished by red and green boxes, respectively. The horizontal lines on the top, bottom, and inside of the box represent the 75% quantile (Q3), 25% quantile (Q1), and median, respectively. The points distributed outside the upper and lower edges represent the outliers of each indicator.

The amino acid, acylcarnitine, and biochemical profiles were detected every 2–3 months and dynamic changes of some typical indicators were analyzed during 1–12 months (Figure 3). The levels of Phe and Tyr elevated to peak levels then declined to normal during ages 1–2 months in both groups, so the data were not drawn. Dynamic change analysis showed that DBil, ALB, Arg, and Cit were the earliest metabolic derangements to reach peak levels in NICCD groups, followed by acylcarnitine profiles (C0, C18:1, and C18:2), and finally with the elevation of liver enzymes (AST and ALT). Overall, the recovery rates of all the abnormal indicators in the “Newborn-screen Group” were faster than those in the “Clinical diagnosed Group.”

For Met, the peak concentrations of the “Newborn-screen Group” and “Clinical diagnosed Group” were later than Arg and Cit, at ages 2–3 months and 4–5 months, respectively. The recovery ages of the majority of patients were at 3–4 months and 6–9 months, respectively.

For GGT, the “Newborn-screen Group” had peak concentrations at 1–3 months and declined to nearly normal levels during 5–6 months, while the “Clinical diagnosed Group” had peak concentrations at 2–3 months and declined to nearly normal levels during 9–12 months.

### Sequential order analysis of typical indicators in the “newborn-screen group” and “clinical diagnosed group” during the recovery phase

**FIGURE 4:**
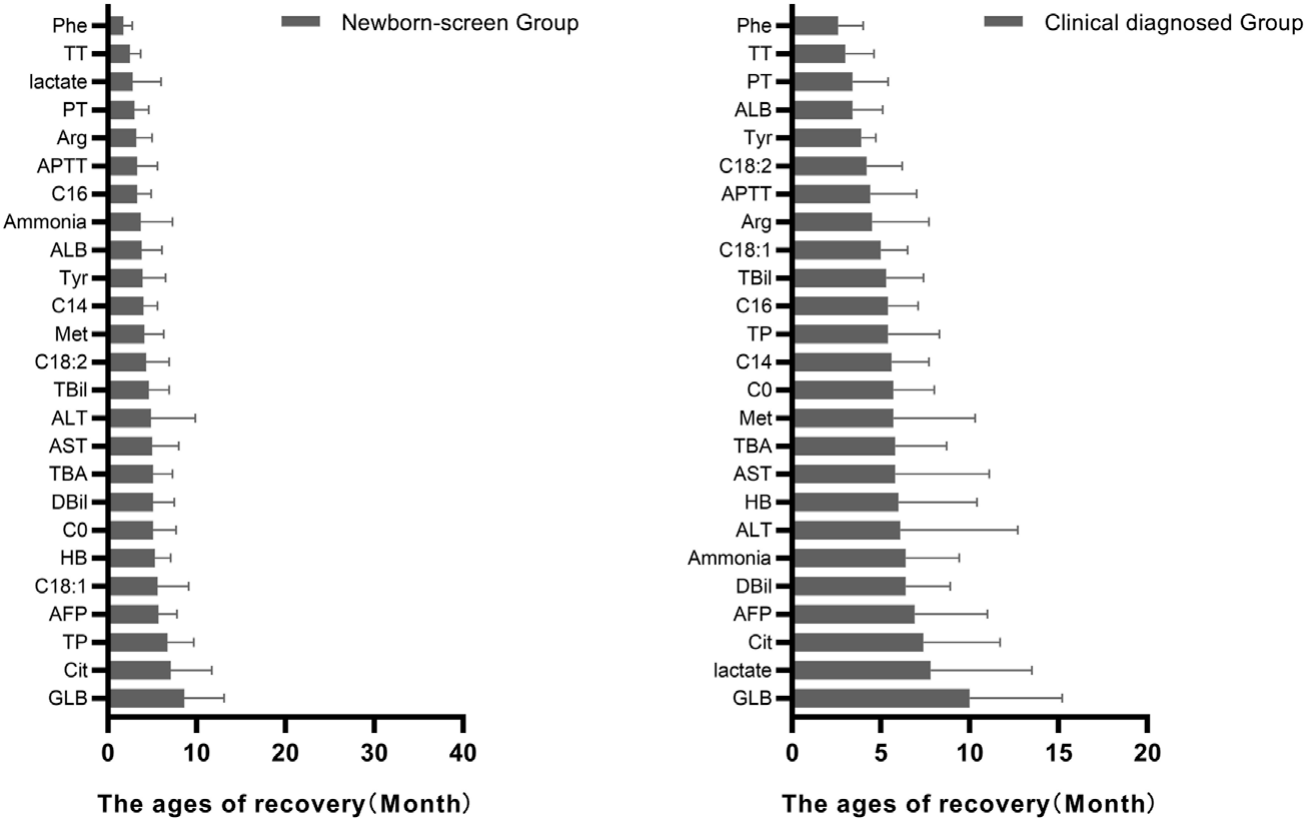
Ages of patients in the “Newborn-screen Group” and “Clinical diagnosed Group” when each indicator starts to return to normal. The ages of recovery of each indicator were described as mean ± standard deviation (SD) and arranged from smallest to largest.

The ages of the typical abnormal indicators to get back to normal were recorded and their sequential orders were analyzed. In the amino acid profiles, Phe was the earliest recovered indicator while Cit was the latest recovered indicator in both groups (Figure 4).

In the acylcarnitine profile, C16 was the earliest recovered indicator while C18:1 was the latest recovered indicator in the “Newborn-screen Group.” C18:2 was the earliest recovered indicator while C0 was the latest recovered indicator in the “Clinical diagnosed Group.”

As for the biochemical data, coagulogram (TT, PT, and APTT) was the early recovered indicator while AFP and GLB were the late recovered indicators in both groups. The lactate was recovered early in the “Newborn-screen Group” while lately recovered in the “Clinical diagnosed Group.”

### The frequency of *SLC25A13* mutations and analysis of genotype–phenotype associations

**FIGURE 5:**
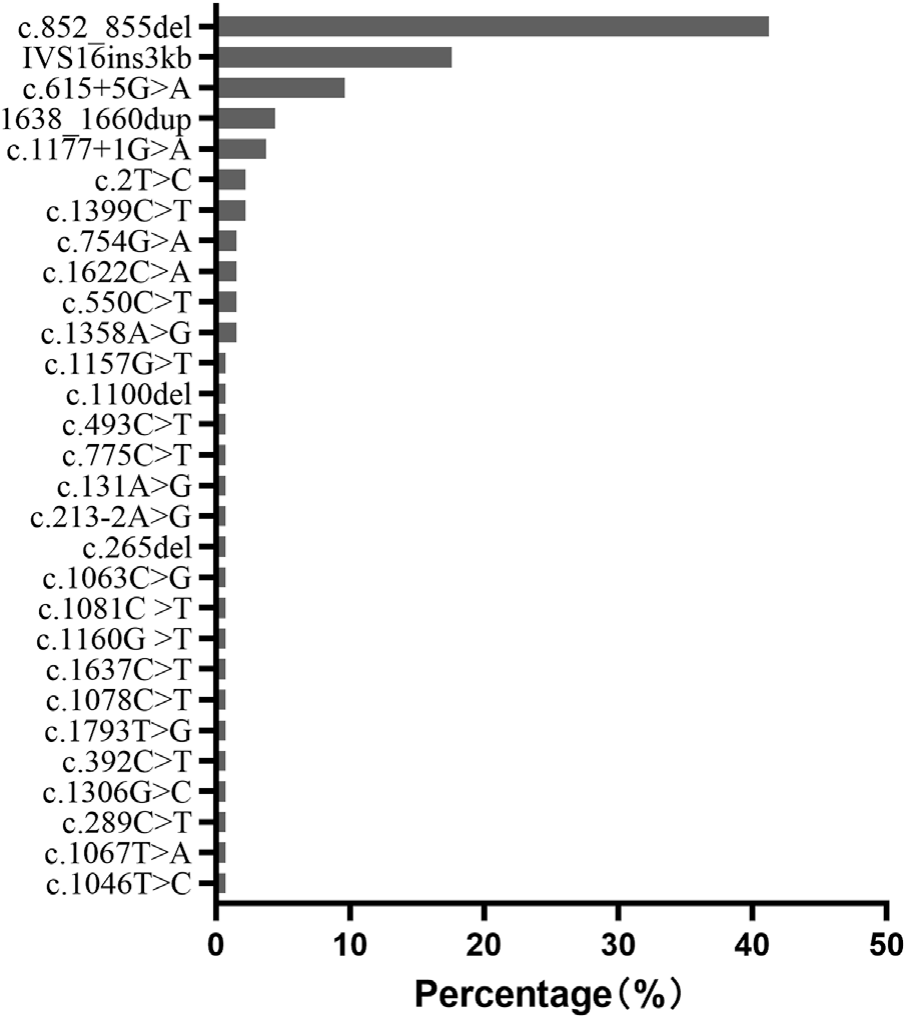
Frequency and proportion of *SLC25A13* mutations in Chinese populations in our population.

c.852_855del (p.Met285Profs*2), IVS16ins3kb, c.615 + 5G>A, 1638_1660dup (p.Ala554Glyfs*17), and c.1177 + 1G>A were the most common mutations with a frequency of 41.2%, 17.6%, 9.6%, 4.4% and 3.7%, respectively. They accounted for 76.5% of all the mutated *SLC25A13* alleles in our population (Figure 5).

## Discussion

Citrin deficiency is recognized as a common cause of infant cholestatic jaundice (Ohura et al., 2007). The estimated frequency of patients with citrin deficiency is 1/38000-7100 in Asia depending on the carrier frequency of pathogenic *SLC25A13* variants (Kobayashi et al., 2003; Tabata et al., 2008; Treepongkaruna et al., 2012; Lin et al., 2020). However, the total frequency of newborn screening with NICCD in our population was 1:82352, similar to the observed prevalence in Japan (1/84,782) (Yamaguchi-Kabata et al., 2019). This indicated that not a few NICCD cases are missed in newborn screening and some of them develop symptoms during infancy. Different from the newborn screened patients who could be timely treated, the NICCD patients diagnosed clinically always have more severe and acute symptoms including cholestasis and hepatic dysfunction. In this study, we studied the differences in metabolic indices and their dynamic changes between the “Newborn-screen Group” and “Clinical diagnosed Group” for a better understanding of the disease.

**FIGURE 6:**
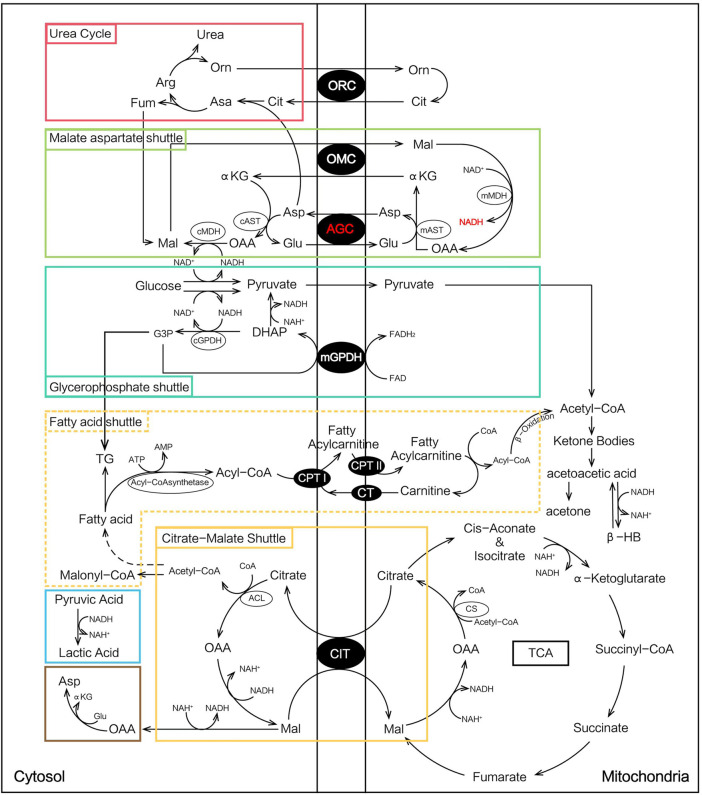
Metabolic pathways involved in NICCD patients.

At the onset of NICCD, we found that the “Clinical diagnosed Group” had significantly higher levels of Cit, Arg, Met, and Orn and lower concentrations of Phe, Ala, Leu, Pro, and Val than the “Newborn-screen Group.” Deficiency of citrin reduced argininosuccinate synthesis and the accumulation of intermediate products of the urea cycle such as Cit, Arg, and Orn were more obvious in the “Clinical diagnosed Group” (Figure 6). The increase of cytosolic NADH/NAD^+^ ratio in citrin deficiency inhibits gluconeogenesis which might have to depend on glucogenic amino acids (Figure 6). In our study, several glucogenic amino acids (Ala, Pro, and Val) were significantly decreased in the “Clinical diagnosed Group,” supporting the enhancement of amino acid gluconeogenesis. Impaired ketogenesis has been reported in CTLN2 (Saheki et al., 2004), but with little evidence in the NICCD stage. Ketogenic/glucogenic amino acid (Phe) and ketogenic amino acid (Leu) were obviously declined in the “Clinical diagnosed Group.” Sequential order analysis (Figure 4) showed that the decline rate of Phe was the fastest in amino acids in both groups. It suggested that Phe might be accelerated to utilize in energy metabolism because of impaired gluconeogenesis and ketogenesis.

In acylcarnitine profiles, the “Clinical diagnosed Group” presented with significantly higher C0, C2, C3, and long-chain acylcarnitines (especially C14, C16, C16:1, C18:1, and C18:2) than the “Newborn-screen Group.” The increased long-chain acylcarnitines had been reported in patients with NICCD during infancy and indicated suppressive β-oxidation of fatty acids (FAO) in the mitochondria (Lee et al., 2006; Xing et al., 2010). In the patients with citrullinemia, the main regulator of fatty acid oxidation, peroxisome proliferator-activated receptor α (PPARα), had been observed to be downregulated (Komatsu et al., 2015). On the other hand, the malate-citrate NADH shuttle is activated by a compensatory mechanism which increased fatty acid synthesis (Lee et al., 2006) (Figure 6). Combining these two aforementioned aspects, the elevation of long-chain acylcarnitines in the “Clinical diagnosed Group” was prominent. However, the concentrations of several medium-chain acylcarnitines were lower in the “Clinical diagnosed Group.” It was reported lower levels of medium-chain acylcarnitines were likely associated with pathologic alterations of an undefined pathway that affected hepatic mitochondrial and/or peroxisomal FAO which eventually led to impaired ketogenesis (Fukao et al., 2004; Ciavardelli et al., 2016).

OPLS-DA showed that Cit, Arg, Met, Orn, Phe, Ala, Leu, Val, C0, C3, C16:1OH, C18:1, and C18:2 contributed most to the discrimination between the “Newborn-screen Group” and “Clinical diagnosed Group.” In a previous study, Ala/Cit used as a secondary indicator can diagnose more confirmed cases and increase positive predictive values (Tang Chenfang et al., 2019). We suggested exploring other secondary ratio indicators of amino acids and/or acylcarnitine may help diagnose additional cases that were missed by using a single indicator of citrulline.

As for biochemical indices, the “Clinical diagnosed Group” has higher levels of bilirubin (DBil), TBA, liver enzymes (ALT, AST, and LDH), ammonia, lactate, TCH, and prothrombin time (TT) than the “Newborn-screen Group.” Bilirubin and TBA were the major elevated indicators of cholestasis. TBA was also one of the most contributed discriminations with the “Newborn-screen Group” showed by OPLS-DA. Dynamic change analysis showed that the age of peak concentration of TBA in the “Clinical diagnosed Group” was 2 months later and the concentration was much higher than that in the “Newborn-screen Group.” It was thought that the severely affected excretion of TBA was the main mechanism of cholestasis in NICCD (Wang et al., 2012). Coagulopathy (especially prolonged TT) is probably caused by malabsorption of vitamin K also due to cholestasis. On the other hand, OPLS-DA showed that AST was a primarily contributed discrimination factor between the two groups. AST levels higher than ALT levels reflected more serious damage of liver cells (Lu et al., 2017). In the “Clinical diagnosed Group,” the concentrations of total protein, amylase, and hemoglobin were lower than in the “Newborn-screen Group.” Hypoproteinemia and reduction of amylase activity also reflected advanced hepatic damage (Donaldson et al., 1979). Overall, we observed that impaired liver function and cholestasis were more serious in the “Clinical diagnosed Group.”

Dynamic change analysis showed that DBil, ALB, Arg, and Cit were the earliest metabolic derangements to reach peak levels in NICCD groups, followed by acylcarnitine profiles, and finally with the elevation of liver enzymes. This seemed to be accordant with previous findings (Lee et al., 2006). Met reached peak levels later than Arg and Cit. The age of its peak concentration in the “Clinical diagnosed Group” was two months later than in the “Newborn-screen Group” while the recovery age was 3–6 months later. Met is the single essential sulfur-containing amino acid and plays important roles in methyl group metabolism, mainly in the liver (Duro et al., 2010). In liver disease, the metabolism of methionine is reduced and the aberrant methyl group flux can further aggravate hepatic dysfunction (Dever and Elfarra, 2010). It suggested that Met could be an indicator of the progression of liver damage.

All characteristic metabolic indicators in the “Newborn-screen Group” came back to normal levels at earlier ages than in the “Clinical diagnosed Group.” We found that in the amino acid profiles, Phe and Tyr (data not shown) were the earliest abnormal indicators in the “Clinical diagnosed Group.” In our previous case, we found an NICCD patient with a suspected diagnosis of phenylketonuria initially (data not shown). Two NICCD screen-negative patients were reported to be misdiagnosed with tyrosinaemia type I (Ohura et al., 2007). These cases suggest that when elevated levels of Phe or Tyr are detected by newborn screening, we should monitor the Cit level to exclude NICCD. Furthermore, Cit was the latest recovered amino acid in both groups which confirmed that it is the most appropriate amino acid indicator to monitor the disease status.

ALB was the earliest abnormal biochemical indicator in both groups. Coagulogram (TT, PT, and APTT) was recovered early while AFP and GLB were the late recovered indicators in both groups. Protein synthesis may be affected by liver dysfunction in NICCD and the immature liver delays the conversion of AFP to ALB (Song et al., 2009). We suggested that ALB could be used as a biochemical indicator for early diagnosis and GLB for prognosis. The lactate was recovered early in the “Newborn-screen Group” while lately restored in the “Clinical diagnosed Group,” which indicated that the resolution of acidosis is faster in the “Newborn-screen Group.” By an analysis of the sequential changes in metabolic indices, we highlighted the importance of newborn screening of NICCD, that rapid detection and treatment can lead to a better prognosis.

In our population, c.852_855del (41.2%), IVS16ins3kb (17.6%), c.615 + 5G>A (9.6%), 1638_1660dup (4.4%), and c.1177 + 1G>A (3.7%) accounted for 76.5% of all the mutated *SLC25A13* alleles. It is consistent with other reports in Chinese cohorts (Song et al., 2013). However, the most prevalent mutation in Japanese NICCD patients is c.1177 + 1G>A (36%), followed by c.852_855del (33%) (Tabata et al., 2008), while in Korean patients, the common pathogenic alleles were IVS16ins3kb (33%), c.852_855del (30%), and c.1177 + 1G>A (12%) (Oh et al., 2017). It seems that Chinese cohorts have higher frequencies of c.852_855del, c.615 + 5G>A, and 1638_1660dup mutations than Japanese and Korean NICCD patients. This phenomenon might be attributed to different founding populations in Asia.

## Data Availability

The datasets presented in this study can be found in online repositories. The names of the repository/repositories and accession number(s) can be found below: “Figshare” and the accession numbers are 10.6084/m9.figshare.20347311, 10.6084/m9.figshare.20347362.
