# Supplementary material for: Dynamic changes of metabolic characteristics in neonatal intrahepatic cholestasis caused by citrin deficiency
Source: Front Mol Biosci. 2022 Aug 24;9:939837. doi: 10.3389/fmolb.2022.939837 (PMC9449879; doi:10.3389/fmolb.2022.939837)
Supplement: Supplementary file 1 [file Table1.docx]

**TABLE S1**  MS/MS results of “Newborn-screen Group” and “Clinical diagnosed Group” at first detection

| **Indices** | **Newborn-screen Group**  **(Average±SD, positive rate)** | **Reference intervals for 3-7days** | **Clinical diagnosed Group**  **(Average±SD, positive rate)** | **Reference intervals for 1-12 months** |
| --- | --- | --- | --- | --- |
| Cit^**^↑^a^ | 148.98±121.67 (100.00%) | 7.14-37 | 279.40±140.92 (100.00%) | 7.18-36.53 |
| Arg^**^↑ | 23.88±23.50 (7.1%) | 2.54-50 | 72.03±44.75 (47.8%) | 0.92-60.59 |
| Met^**^↑ | 37.38±37.37 (26.2%) | 7.18-41.35 | 146.45±145.52 (58.4%) | 7.96-55.40 |
| Phe^**^↑ | 98.16±53.09 (38.10%) | 23.3-100 | 55.37±42.39 (2.6%) | 22.14-101.40 |
| Tyr^*^↑ | 193.05±101.25 (21.43%) | 34.5-280 | 152.81±81.48 (46%) | 25.90-151.84 |
| Ala^**^↓^b^ | 228.34±118.94 (2.4%) | 136.5-650 | 208.95±87.68 (2.6%) | 98.38-556.89 |
| Gly^**^↓ | 409.16±151.12 (4.76%) | 246.57-1283 | 263.01±125.05 (0.1%) | 116.64-556.89 |
| Leu^**^↓ | 181.11±55.22 (2.38%) | 75.7-316 | 118.76±41.69 (3.5%) | 59.00-342.87 |
| Orn^**^↑ | 167.82±67.33 (2.38%) | 52.09-386.6 | 222.74±78.25 (43.3%) | 35.68-223.20 |
| Pro^**^↑ | 258.37±127.65 (4.76%) | 97.2-440.3 | 203.90±88.83 (4.4%) | 61.72-316.22 |
| Val^**^↓ | 179.30±55.57 (0%) | 51.7-305 | 122.23±37.79 (0%) | 55.03-335.31 |
| C0^**^↑ | 32.31±16.29 (4.76%) | 10.28-54.24 | 71.44±29.84 (23.9%) | 12.02-87.19 |
| C2^**^↑ | 17.76±7.64 (0%) | 3-50 | 26.71±11.14 (2.59%) | 5.47-51.79 |
| C3^**^↑ | 1.85±0.70 (0%) | 0.43-3.8 | 3.94±3.76 (18.5%) | 0.35-5.26 |
| C4DC+C5OH↑ | 0.22±0.0 6 (0%) | 0.07-0.48 | 0.29±0.09 (0%) | 0.11-0.68 |
| C6^*^↓ | 0.05±0.03 (7.14%) | 0.03-0.17 | 0.07±0.05 (6.03%) | 0.02-0.15 |
| C6DC^**^↓ | 0.08±0.06 (16.67%) | 0.04-0.28 | 0.05±0.03 (0%) | 0.02-0.22 |
| C8↓ | 0.07±0.05 (0%) | 0.02-0.17 | 0.06±0.05 (2.6%) | 0.02-0.21 |
| C8:1^**^↓ | 0.11±0.06 (7.14%) | 0.05-0.43 | 0.08±0.04 (8%) | 0.04-0.60 |
| C10↓ | 0.08±0.08 (0%) | 0.03-0.22 | 0.06±0.03 (0%) | 0.02-0.28 |
| C10:1↓ | 0.07±0.05 (16.67%) | 0.04-0.27 | 0.06±0.03 (1.7%) | 0.02-0.24 |
| C10:2↓ | 0.01±0.01 (2.38%) | 0.01-0.08 | 0.02±0.02 (0%) | 0-0.04 |
| C14^**^↑ | 0.21±0.12 (7.14%) | 0.07-0.4 | 0.37±0.17 (37.93%) | 0.03-0.33 |
| C14:2^*^↑ | 0.02±0.02 (2.38%) | 0.01-0.06 | 0.04±0.04 (18.1%) | 0.01-0.07 |
| C16^**^↑ | 2.51±1.55 (4.76%) | 0.49-6 | 3.85±1.84 (59.2%) | 0.29-3.22 |
| C16:1^**^↑ | 0.15±0.11 (0%) | 0.02-0.49 | 0.2±0.1 (35.3%) | 0.02-0.22 |
| C16:1OH^**^↑ | 0.04±0.03 (4.76%) | 0.01-0.1 | 0.07±0.04 (18.1%) | 0.01-0.10 |
| C18^**^↑ | 0.75±0.34 (0%) | 0.24-1.9 | 0.92±0.47 (20.3%) | 0.13-1.24 |
| C18:1^**^↑ | 1.41±0.52 (2.38%) | 0.38-2.92 | 2.47±1.08 (31.8%) | 0.26-2.88 |
| C18:2^**^↑ | 0.39±0.28 (2.38%) | 0.07-0.8 | 1.15±0.51 (55.8%) | 0.08-0.95 |

Abbreviations: ^**^, means the difference in two groups is significant at the 0.01 level; ^*^, means the difference in two groups is significant at the 0.05 level; ↑^a^, means the positive rate is calculated for the values higher than the normal range; ↓^b^, means the positive rate is calculated for the values lower than the normal range; Cit, citrulline; Arg, arginine; Met, methionine; Phe, phenylalanine; Tyr, tyrosine; Ala, alanine; Gly, glycine; Leu, leucine; Orn, ornithine; Pro, proline; Val, valine.
